# The impact of intraoperative MRI on cranial surgical site infections—a single-center analysis

**DOI:** 10.1007/s00701-023-05870-6

**Published:** 2023-11-16

**Authors:** Ann-Kathrin Joerger, Jimmy E. Laho, Victoria Kehl, Bernhard Meyer, Sandro M. Krieg, Sebastian Ille

**Affiliations:** 1 Department of Neurosurgery, Technical University of Munich, Klinikum rechts der Isar, Ismaningerstr. 22, 81675 Munich, Germany; 2 Institute for AI and Informatics in Medicine, Technical University of Munich, Ismaninger Str. 22, 81675 Munich, Germany

**Keywords:** Surgical site infections, Cranial SSI, ioMRI, Intraoperative MRI

## Abstract

**Purpose:**

The use of intraoperative MRI (ioMRI) contributes to an improved extent of resection. Hybrid operating room MRI suites have been established, with the patient being transferred to the MRI scanner. In the present descriptive analysis, we compared the rate of surgical site infections (SSI) after intracranial tumor surgery with and without the use of ioMRI.

**Methods:**

In this retrospective study, we included 446 patients with open craniotomy performed for brain tumor surgery. One hundred fourteen patients were operated on with the use of ioMRI between June 1, 2018, and June 30, 2019 (group 1). During the same period, 126 patients were operated on without ioMRI (group 2). As an additional control group, we analyzed 206 patients operated on from February 1, 2017, to February 28, 2018 when ioMRI had not yet been implemented (group 3).

**Results:**

The rate of SSI in group 1 (11.4%), group 2 (9.5%), and group 3 (6.8%) did not differ significantly (*p* = 0.352). Additional resection after ioMRI did not result in a significantly elevated number of SSI. No significant influence of re-resection, prior radio-/chemotherapy, blood loss or duration of surgery was found on the incidence of SSI.

**Conclusion:**

Despite the transfer to a non-sterile MRI scanner, leading to a prolonged operation time, SSI rates with and without the use of ioMRI did not differ significantly. Hence, advantages of ioMRI outweigh potential disadvantages as confirmed by this real-life single-center study.

**Supplementary Information:**

The online version contains supplementary material available at 10.1007/s00701-023-05870-6.

## Introduction

Surgical site infections (SSI) are of profound clinical importance. Rates of SSI in the field of neurosurgery vary from 1.2% [19] to 8.0% [17, 20]. SSI are a burden not only to the health care system generating additional costs [21], but also to the patients leading to readmission or a prolonged postoperative hospital stay, reoperation and an increased mortality [7, 16]. Cancer patients harbor an elevated risk for developing SSI because they are often immunocompromised [2]. It is recommended to begin radiotherapy a minimum of 7 days after surgery so as not to hamper the early phases of wound healing [14]. Consequently, SSI lead to a delay in adjuvant radiotherapy.

In brain tumor surgery, the use of intraoperative MRI (ioMRI) has gained increasing importance in recent years, contributing to an improved extent of resection [3, 10, 13, 15]. For glioma as well as for intracranial metastases it has been shown that a gross total resection is associated with an improved outcome [4, 22, 24]. IoMRI not only helps to visualize remaining tumor tissue but can also update neuronavigation to the intraoperative situation.

An MRI scanner within the operating room requires that all surgical instruments and anaesthesiologic equipment be MRI-compatible [12], which is difficult to realize. Therefore, dual independent operating room MRI suites have been established, with the patient being transferred from the operating table to the MRI, which is in a separated room close by [5, 12, 18].

However, the use of ioMRI prolongs the duration of the operation by 78 min on average [12]. A prolonged surgical time is a known risk factor for SSI [6]. Moreover, for the transfer of the patient to the MRI suite, the operation site must be draped [5], bearing the risk for contamination of the sterile field. Previous studies analyzing the rate of SSI after craniotomies using a dual operating room MRI suite found rates within the normal range [8, 23]. Yet, these studies did not report on a control group of patients being operated on without the use of ioMRI in the same setting. With this study, we aim to compare the rates of SSI after brain tumor surgery with and without the use of ioMRI by a descriptive analysis in a real-world scenario.

## Methods

### Study design

We conducted a retrospective analysis comprising patients undergoing intracranial tumor resection at the department of neurosurgery of a tertiary care hospital from February 1, 2017, to June 30, 2019. Patients with gliomas as well as metastases were included. To eliminate a bias due to differences of the surgical approach stereotactic biopsies, endoscopic and transnasal approaches and scull base tumors were excluded. To minimize the risk of bias from changing surgical teams and other time dependent influences, patients were divided into three groups: Patients being operated on between June 1, 2018, and June 30, 2019, with the use of ioMRI were included in group 1. Group 2 comprised patients being operated during the same period without ioMRI. Group 3 was formed by patients operated on from February 1, 2017, to February 28, 2018, when ioMRI had not yet been implemented at all. Rates, types, risk factors, microbiological spectrum and treatment of SSI were analyzed.

### The use of ioMRI and technical details

Since March 1, 2018 ioMRI has been used routinely for all glioma operations in our department. For metastases it is used if, based on preoperative imaging, a glioma cannot be excluded or if total resection is challenging because of an eloquent location. The study started on June 1, 2018 to exclude the bias of adaptation of surgical routine to a new setting.

Our department uses a two-room ioMRI setup (3T MR scanner Ingenia, Philips Medical System, Netherlands B.V.) and an MRI-compatible head clamp, including an 8-channel coil array (Noras MRI products, Hoechberg, Germany). The MRI room and the operating room are separated from each other through a sliding door. The MRI room has an additional access from outside the operation theater for outpatients. Therefore, the scanner room is cleaned 40 min before each ioMRI scan. Anesthesiologic monitoring equipment is MRI-compatible. For transfer to the MRI-scanner neuromonitoring and magnetic operating equipment is removed. After the initial resection and hemostasis, the cavity is refilled with ringer’s solution. The surgical site is provisionally closed with a collagen sponge including rough sutures and covered with sterile gauze dressing pads and an incision drape (supplementary table 1). Then the patient’s head is draped completely in a sterile manner. The scanner room is locked to the outpatient region and is cleaned. Before the transfer a checklist is filled out to ensure that all magnetic items have been removed from the patient [8]. For re-resection, neuromonitoring is once again connected to the patient. For this purpose, the stimulation electrodes are applied under sterile conditions. IoMRI data is analyzed by a neuroradiologist and a board neurosurgeon. If there is residual tumor depicted and an additional resection is feasible it is performed. IoMRI data is used to update the neuronavigation data using Brainlab (Munich, Germany) software.

### Antibiotic prophylaxis

All patients receive an antibiotic prophylaxis of single-shot cefuroxime 1.5 g intravenously prior to skin incision followed by another dose if the operation takes longer than 4 h. In an event of an allergy, clindamycin is used.

### Definition of surgical site infections

Surgical site infections were classified as superficial, deep, epidural and intracranial (empyema, abscess), meningitis/ventriculitis, infected cerebrospinal fluid (CSF) fistula and shunt infection. Isolated CSF fistulas were excluded.

### Statistical analysis

Patients’ baseline characteristics and surgical details were compared using a chi-square test, a Fisher’s exact test, a Kruskall-Wallis test and a Mann-Whitney-*U* test adjusted for multiple testing. To compare the rate of SSI and the different types of SSI between the three groups, a chi-square test and Fisher’s exact test were used. To evaluate the influence of different risk factors on SSI multivariate logistic regression analysis was performed.

Statistical analysis was performed using IBM SPSS Statistics version 26 (Armonk, New York, USA).

## Results

### Patient cohorts and characteristics

In total, 446 patients who underwent open surgery for glioma or an intracranial metastasis were included. One hundred fourteen patients were in group 1, 126 in group 2 and 206 in group 3 (Table 1). The median age was significantly different between the three groups (*p*=0.001) (Table 1). As ioMRT is mainly used for glioma operations, in group 1 almost all cases were gliomas (91.2%), whereas in group 2 metastases were more frequent (65.1%) (Table 1). In group 3, more patients suffered from gliomas (60.7%) than from metastases (39.3%). The majority of surgeries were first-time operations. However, the three groups differed significantly regarding the rate of operations for tumor recurrence (*p*<0.001) (Table 1).

**Table 1:** Patients’ characteristics

	Group1 (*n* = 114)		Group 2 (*n* = 126)		Group 3 (*n* = 206)		*p*-value
Sex, *n (%)*							0.806^*^
Male	68	(59.6%)	74	(58.7%)	128	(62.1%)	
Female	46	(40.4%)	52	(41.3%)	78	(37.9%)	
Age (years), *median (range)*	58	(18 – 88)	66	(20 – 89)	59	(10 – 89)	0.001^#^ Adj. Sig^§^: 1 – 3: 1.000 1 – 2: < 0.001 2 – 3: < 0.001
Diagnosis, *n (%)*							< 0.001^*^
Glioma	104	91.2%	44	34.9%	125	60.7%	
Metastasis	10	8.8%	82	65.1%	81	39.3%	
Recurrent tumor, *n (%)*							< 0.001^*^
no	80	70.2%	116	92.1%	169	82.0%	
yes	34	29.8%	10	7.9%	37	18.0%	

^*^ *χ2 test (or Fisher exact test)*

*# Kruskall-Wallis test*

*§ Pairwise comparisons with the Mann-Whitney-U test, adjusted for multiple testing*

Group 1: Patients operated on between June 1, 2018, and June 30, 2019, by ioMRI (mostly gliomas)

Group 2: patients operated during the same period without ioMRI (mostly metastases)

Group 3: control cohort with patients operated on from February 1, 2017, to February 28, 2018, when ioMRI had not been implemented

### Surgical details

The median time of surgery was significantly longer when using ioMRI compared to operations without it (*p*<0.001) (Table 2). Median intraoperative blood loss did not differ significantly (*p*=0.220).

**Table 2:** Surgical details

	Group1		Group 2		Group 3		*p*-value
	(*n* = 114)		(*n* = 126)		(*n* = 206)		
Duration of surgery (min), *median (range)*	245	(74 – 453)	134	(48 – 334)	174	(23 – 459)	<0.001^#^ Adj. Sig^§^: 1 – 3: < 0.001 1 – 2: < 0.001 2 – 3: < 0.001
Intraoperative blood loss (ml), *median (range)*	(*n* = 96)		(*n* = 107)		(*n* = 180)		0.220^#^
	400	(70 –2000)	300	(50 – 1970)	400	(50 – 2867)	
Additional resection after ioMRI, *n (%)*	(*n* = 111)						
no	79	71.2%					
yes	32	28.8%					

*# Kruskall-Wallis test*

*§ Pairwise comparisons with the Mann-Whitney-U test, adjusted for multiple testing*

Group 1: Patients operated on between June 1, 2018, and June 30, 2019, by ioMRI (mostly gliomas)

Group 2: patients operated during the same period without ioMRI (mostly metastases)

Group 3: control cohort with patients operated on from February 1, 2017, to February 28, 2018, when ioMRI had not been implemented

**Fig. 1:**
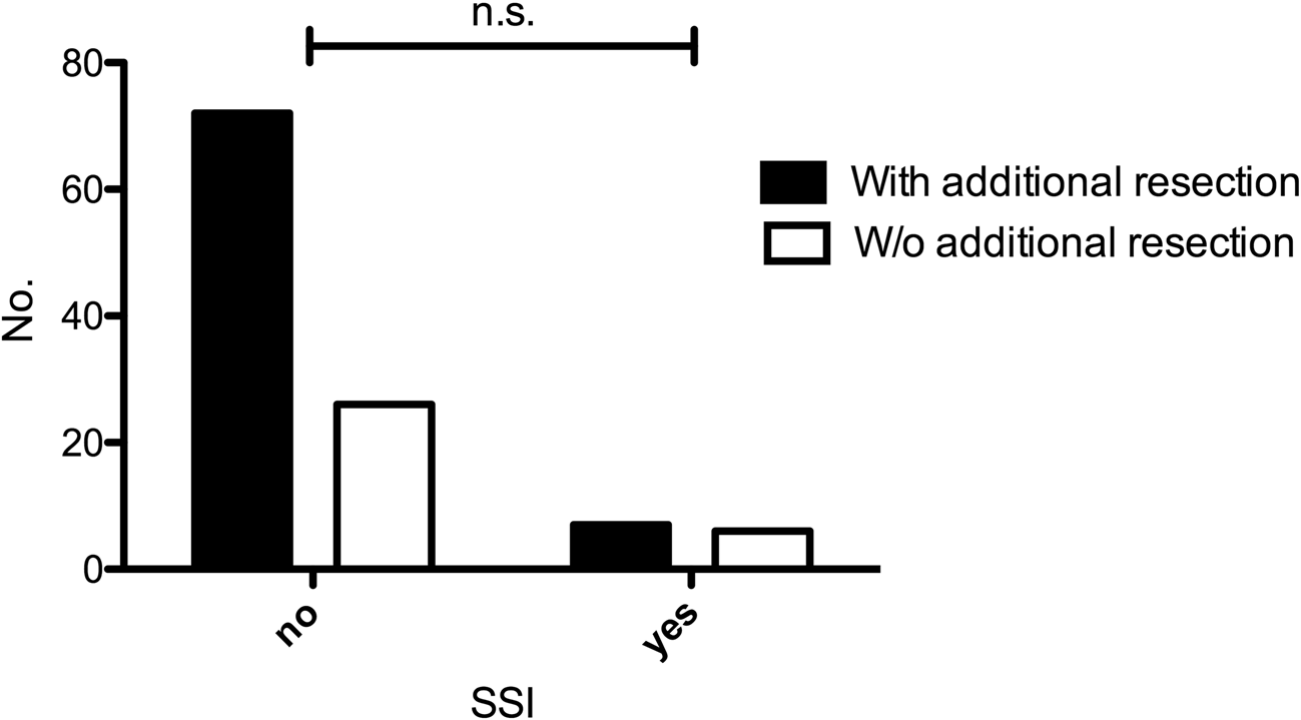
SSI with additional resection after ioMRI. This figure depicts the absolute number of SSI depending on the resection status in group 1. Group 1: Patients operated on between June 1 2018 and June 30 2019 by ioMRI (mostly gliomas). SSI = surgical site infections. *p*=0.192, χ^2^ test (or Fisher exact test)

In 32 cases (28.8%) an additional resection after ioMRI was performed (Table 2). SSI rates of these patients did not differ significantly from those without additional resection (*p*=0.192) (Fig. 1).

### Rate of surgical site infections

The difference between the rates of SSI in group 1 (11.4%, 95% CI 6.8–18.5%), group 2 (9.5%, 95% CI 5.5–15.9%), and group 3 (6.8%, 95% CI 4.1–11.1%) was statistically not significant (*p* = 0.352) Table 3).

**Table 3:** Details of surgical site infections

	Group 1 (*n*=114)	Group 2 (*n*=126)	Group 3 (*n*=206)	*p*-value
Total SSI	13 (11.4%)	12 (9.5%)	14 (6.8%)	0.352*
95% Confidence Intervall Type of SSI	6.8 – 18.5%	5.5 – 15.9%	4.1 – 11.1%	
Superficial	3	1	1	0.260*
Deep	2	3	2	
Intracranial (empyema, abscess)	3	4	5	
Ventriculitis/meningitis	3	2	6	
Infected CSF fistula	2	1	0	
Shunt infection	0	1	0	

* χ^*2*^ *test (or Fisher exact test)*

Group 1: Patients operated on between June 1, 2018, and June 30, 2019, by ioMRI (mostly gliomas)

Group 2: Patients operated during the same period without ioMRI (mostly metastases)

Group 3: control cohort with patients operated on from February 1, 2017, to February 28, 2018, when ioMRI had not been implemented

To analyze the influence of common risk factors on the rate of SSI multivariate logistic regression analysis was performed. No significant influence of resection for tumor recurrence, prior radiotherapy, prior chemotherapy ever, prior chemotherapy during the last three months, intraoperative blood loss and duration of surgery on SSI was found (supplementary table 2). Moreover, the type of diagnosis did not have a significant influence on SSI.

### Types of surgical site infections and microbiological report

The most frequent types of SSI in group 1 were superficial infections (3 out of 13), intracranial infections (3) and ventriculitis/meningitis (3) (Table 3). In group 2, intracranial infections (4 out of 12) were followed by deep SSI (3). In group 3, ventriculitis/meningitis (6 out of 14) was followed by intracranial infections (5). There was no significant difference between the types of SSI when the three groups were compared (*p* = 0.260). Revision surgery for SSI was performed in nine out of 13 (69.2%) patients in group 1, in ten out of twelve (83.3%) patients in group 2 and in eight out of 14 (57.1%) patients in group 3 (Table 4). In total, in twelve cases more than one revision surgery due to an SSI was necessary. Bone flap removal was necessary in two out of 13 (15.3%) patients in group 1, in three out of twelve (26.0%) patients in group 2 and in six out of 14 (42.9%) in group 3.

**Table 4:** Surgical procedures following SSI

	Group 1 (*n*=13)	Group 2 (*n*=12)	Group 3 (*n*=14)
Revision surgery	9 (69.2%)	10 (83.3%)	8 (57.1%)
> = 1 revision surgery	2 (15.3%)	7 (58.3%)	3 (21.4%)
removal of bone flap	2 (15.3%)	3 (26.0%)	6 (42.9%)

Group 1: Patients operated on between June 1, 2018, and June 30, 2019, by ioMRI (mostly gliomas)

Group 2: Patients operated during the same period without ioMRI (mostly metastases)

Group 3: control cohort with patients operated on from February 1, 2017, till February 28, 2018, when ioMRI had not been implemented

The most frequent bacteria detected in group 1 and 2 was *Cutibacterium acnes* (Table 5), whereas in group 3 *Staphylococcus aureus* was found most frequently (Table 5). Antibiotic therapy was prescribed to all cases except one case with superficial SSI with negative bacterial cultivation.

**Table 5:** Bacterial spectrum

	Group 1 (*n*=13)	Group 2 (*n*=12)	Group 3 (*n*=14)
Anaerococcus murdochii			1
Bacteroides cellulosilyticus			1
Bacteroides vulgatus			1
Cutibacterium acnes	5	3	2
E. coli			2
E. coli 3 MRGN	1		
Enterobacter cloacae			1
Enterococcus faecalis	1	1	
Finegolida magna			2
Morganella morganii			1
Peptoniphilus tyrrelliae			1
Staph. aureus (MSSA)	1	2	4
Staph. capitis			1
Staph. epidermidis	3	2	2
Staph. lugdunensis			1
Streptococcus constellatus		1	
Streptococcus mitis		1	
Streptococcus oralis			1
No germ	4	4	4

Group 1: patients operated on between June 1, 2018, and June 30, 2019, by ioMRI (mostly gliomas)

Group 2: patients operated during the same period without ioMRI (mostly metastases)

Group 3: control cohort with patients operated on from February 1, 2017, to February 28, 2018, when ioMRI had not been implemented

E. = *Eschericha,* Staph. = *Staphylococcus*

## Discussion

### With and without ioMRI and after additional resection

In this study, we were able to demonstrate that there was no statistically significant difference in the rate of SSI after surgery for intracranial glioma or metastases whether or not the operation was performed using an ioMRI. The rate of SSI slightly increased over the course of time. However, this was statistically not significant. In group 3, an SSI rate of 6.8% was registered. This is in line with the rates described in previous literature (1.2–8.2%) [8, 17, 20]. One reason for the increasing number could be the increase of patients with one or more chronic diseases in general [9]. Multimorbid patients are at a higher risk for complications and hospitalization [9, 11].

Another reason for the comparably high rates of SSI in this study is the fact that contrary to most other authors [16, 20] we did not limit the time period of SSI occurrence and we included meningitis/ventriculitis. Notably, in group 1, there were fewer cases when bone flap removal was necessary and more superficial SSI, indicating less severe infections than in the other groups. In previous studies, SSI rates of craniotomies vary from 4.3 to 8.2% [1, 8, 17]. In these studies, revision surgery because of a SSI was necessary in 45.0 to 73.9% of cases. In our study, rates of revision surgery for SSI were in the upper range.

Additional resection after ioMRI was performed in a relevant number of cases (almost 30%), demonstrating its importance for surgical strategy. Despite additional resection resulting in a prolonged operation time there was no significant difference in SSI rate.

### Further risk factors for SSI

As expected, median operation time was significantly longer using ioMRI. However, by logistic regression, no significant influence of operation time on SSI was shown. Other known risk factors such as intraoperative blood loss, prior operation and prior radio- or chemotherapy had no significant influence either. This finding was contrary to what was described before by McCutcheon *et al.* [16]. They analyzed risk factors for SSI after craniotomy for brain tumor in 12,021 patients. Operation time longer than 4 h and recent chemotherapy were associated with increased odds of SSI.

### Microbiological spectrum

The most frequent bacteria detected in our study were *Cutibacterium acnes* and *Staphylococcus aureus. Cutibacterium acnes* was the most frequent independently from the use of ioMRI. As these bacteria have been previously described as the most frequent bacteria causing SSI after craniotomies [1, 17], the use of ioMRI does not lead to a new microbiological spectrum.

### Limitations

This is a retrospective study. The groups were not randomized with respect to diagnosis, age, recurrent tumor, and prior oncological treatment. Moreover, patient-specific factors such as steroid use, comorbidities and nutritional and smoking status were not analyzed at all. Further prospective randomized studies comprising these factors are needed. This is a single-center study. In other centers, there may be differences regarding the protocols for ioMRI transfer of patients.

SSI rate increased over the course of time. However, the increase was not statistically significant.

### Strengths

This study comprises a large cohort of consecutive patients in a high-volume tertiary oncological center using two control groups. Thus, the results should be regarded as a guidance for future use of ioMRI.

## Conclusion

Despite the transfer to a non-sterile MRI scanner and a consequently prolonged surgical time SSI rates with and without the use of ioMRI did not differ significantly. Moreover, additional resection after ioMRI did not result in significantly increased SSI. As the use of ioMRI led to a relevant number of additional resections and the degree of tumor resection is linked to overall survival in glioma patients, we recommend the use of ioMRI for these tumor entities.

## Supplementary information

Supplementary Table 1
(17.1 KB)

Supplementary Table 2
(16.9 KB)

## Data Availability

Further and anonymized data will be made available upon reasonable request.
