# Supplementary material for: The impact of intraoperative MRI on cranial surgical site infections—a single-center analysis
Source: Acta Neurochir (Wien). 2023 Nov 16;165(12):3593–9. doi: 10.1007/s00701-023-05870-6 (PMC10739228; doi:10.1007/s00701-023-05870-6)
Supplement: Supplementary file 1 — Supplementary Table 1 (17.1 KB) [file 701_2023_5870_MOESM1_ESM.docx]

| Layer | Type |
| --- | --- |
| 1 | Collagen sponge |
| 2 | Sutures |
| 3 | Gauze |
| 4 | Incision drape |
| 5 | Surgial drapes |
|  |  |

**Supplementary table 1: Cover of surgical field for transfer**
