# Supplementary material for: The impact of intraoperative MRI on cranial surgical site infections—a single-center analysis
Source: Acta Neurochir (Wien). 2023 Nov 16;165(12):3593–9. doi: 10.1007/s00701-023-05870-6 (PMC10739228; doi:10.1007/s00701-023-05870-6)
Supplement: Supplementary file 2 — Supplementary Table 2 (16.9 KB) [file 701_2023_5870_MOESM2_ESM.docx]

|  | B | p-value | OR | 95% CI for OR | |
| --- | --- | --- | --- | --- | --- |
|  |  |  |  | Lower | Upper |
| Group |  | 0.452 |  |  |  |
| Group 1 vs. Group 3 | 0.386 | 0.415 | 1.470 | 0.582 | 3.713 |
| Group 2 vs. Group 3 | 0.552 | 0.247 | 1.737 | 0.682 | 4.424 |
| Age at OP (years) | -0.010 | 0.375 | 0.990 | 0.967 | 1.013 |
| Diagnosis (gliom vs. metastases) | 0.055 | 0.913 | 1.057 | 0.391 | 2.860 |
| Re-Resection (yes vs. no) | 0.839 | 0.221 | 2.314 | 0.604 | 8.865 |
| Radiotherapy (yes vs. no) | -0.189 | 0.810 | 0.828 | 0.177 | 3.871 |
| Chemotherapy (yes vs. no) | -0.988 | 0.099 | 0.372 | 0.115 | 1.205 |
| Blood loss (ml) | 0.001 | 0.070 | 1.001 | 1.000 | 1.002 |
| Duration of surgery (min) | 0.000 | 0.965 | 1.000 | 0.994 | 1.006 |
| Constant | -2.377 | 0.017 | 0.093 |  |  |

**Supplementary table 2: Influence of common risk factors on SSI (multivariate logistic regression analysis)**

Group 1: Patients operated on between June 1, 2018, and June 30, 2019, by ioMRI (mostly gliomas);

Group 2: patients operated during the same period without ioMRI (mostly metastases);

Group 3: control cohort with patients operated on from February 1, 2017, till February 28, 2018, when ioMRI had not been implemented.
